# High-Fidelity Modeling of Laser Levels via Pulse-Window Software Lock-In PSD Sensing

**DOI:** 10.3390/s26041180

**Published:** 2026-02-11

**Authors:** Shudong Zhuang, Jiale Sun, Rugao He, Ying Zou, Libin Li, Yu Wan, Ao Sheng

**Affiliations:** 1 College of Mechanical and Electrical Engineering, Hohai University, Changzhou 213251, China; zhsd1970@126.com (S.Z.); 231619010136@hhu.edu.cn (Y.Z.); 2205020204@hhu.edu.cn (L.L.); 231619010090@hhu.edu.cn (Y.W.); 231619010079@hhu.edu.cn (A.S.); 2 Laisai Laser Technology Co., Ltd., Changzhou 213100, China; 13775139113@139.com

**Keywords:** laser level, PSD sensing, software lock-in detection, dynamic identification, high-fidelity modeling

## Abstract

Accurate identification of dynamic parameters, specifically natural frequency and damping ratio, is critical for optimizing the disturbance rejection performance of laser level self-leveling mechanisms. However, traditional Finite Element Analysis (FEA) often struggles to quantify micro-friction damping, while contact measurement methods introduce added mass interference. To address these challenges, this paper proposes an integrated framework combining Pulse-Window Software Lock-in (PWSL) sensing with a data-driven model updating strategy. Initially, a rigid-body dynamic model theoretically predicted a natural frequency (*f*_sim_) of 2.987 Hz and a damping ratio (*ζ*_sim_) of 0.1255. To acquire authentic responses, a non-contact Position Sensitive Detector (PSD) system was developed. The custom PWSL algorithm leverages the laser’s 10 kHz carrier to extract high-fidelity displacement signals, effectively suppressing broadband noise despite embedded hardware limitations. Experimental results demonstrated that the measured frequency (*f*_exp_ = 2.861 Hz) aligned well with predictions (4.22% error). In contrast, the measured damping ratio (*ζ*_exp_ = 0.1435) exceeded the simulation value by 14.34%, quantitatively revealing the energy dissipation caused by unmodeled bearing friction. Based on this disparity, the FEA model was inversely updated by introducing an equivalent friction coefficient, successfully reducing the damping prediction error to 0.97%. This study establishes a high-fidelity updated model, providing a reliable basis for the refined design of precision pendulum instruments.

## 1. Introduction

Precision physical pendulums serve as the core sensing elements in a wide array of modern engineering devices, with applications ranging from gravimeters and laser levels to gyroscopes [1]. The dynamic characteristics of these mechanisms, particularly the natural frequency and damping ratio, directly dictate critical performance metrics such as response speed, stability, and disturbance rejection capability. Consequently, the precise identification and evaluation of these dynamic parameters are indispensable for the structural optimization and performance prediction of such instruments.

As a robust numerical simulation tool, Finite Element Analysis (FEA) has been widely adopted for the dynamic modeling and performance prediction of diverse mechanical systems. Huo et al. employed Finite Element Analysis (FEA) to investigate the dynamic characteristics of a novel stick-slip rotary platform, providing critical theoretical guidance for the design and optimization of its flexible driving mechanism [1]. Lin et al. proposed a novel strategy to implement a complex biomechanical model into a standard FE environment. Their work successfully transferred the damped bipedal inverted pendulum model into ANSYS, enabling the analysis of the dynamic foot-structure contact [2]. Hosseini-Pishrobat and Tatar developed a mathematical model, validated against FEM, to analyze how manufacturing imperfections affect the frequency response of MEMS gyroscopes [3]. These studies highlight the power of simulation in predicting the dynamic behavior of complex mechatronic systems. However, any theoretical model is an approximation of physical reality. Its predictive accuracy, particularly for complex damping mechanisms involving multi-physics coupling such as eddy currents and bearing friction, must be rigorously validated and calibrated against experimental data. For instance, the work by Shi et al. revealed that the behavior of eddy current dampers is highly frequency-dependent, a non-linear characteristic that is challenging to capture accurately with standard finite element models alone [4].

In terms of experimental measurement, traditional contact-based methods inevitably alter the dynamic characteristics of the pendulum body due to the added mass effect. Non-contact dynamic measurement techniques have rapidly advanced in recent years to overcome the interference issues of traditional contact-based methods. Among them, machine vision-based approaches, combining high-speed cameras with image processing, can now track complex motions. Hill et al. successfully utilized Digital Image Correlation (DIC) as a non-contact alternative to traditional strain gages for vibration analysis, though its accuracy can be influenced by factors like camera frame rate and image resolution [5]. In contrast, the Position-Sensitive Detector (PSD) has shown immense potential in fields such as micro-angle measurement, vibration analysis, and high-speed motion tracking, owing to its advantages of high temporal and spatial resolution [6]. Das et al. recently developed a complete measurement system where a PSD, integrated with laser triangulation, was utilized to capture high-fidelity vibration data [7]. Sun et al. leveraged these characteristics to develop a high-resolution position and angle measurement system, demonstrating the PSD’s critical role in achieving high-precision applications [8]. In sensor signal processing, traditional Analog Lock-in Amplifiers (LIA) offer superior noise rejection capabilities; however, their bulky size and high cost render them unsuitable for integrated applications [9]. With the advancement of digital signal processing, techniques such as the Discrete Wavelet Transform (DWT) have been widely applied in sensor data acquisition due to their ability to handle non-stationary transient signals. For instance, Julian et al. investigated a deep-wavelet neural network approach to calibrate low-cost sensor devices, demonstrating that advanced signal processing can effectively mitigate noise interference and significantly enhance measurement accuracy [10]. However, the implementation of complex high-order algorithms on embedded microcontrollers (MCU) is constrained by limited computational resources, whereas conventional digital filters often introduce detrimental phase lags [11]. Therefore, achieving high Signal-to-Noise Ratio (SNR) extraction of weak signals within low-cost hardware architectures remains a technical challenge.

Despite significant advancements in both theoretical simulation and experimental measurement, a comprehensive research framework that deeply integrates high-precision sensing with high-fidelity modeling remains lacking for precision physical pendulums, such as the self-leveling mechanisms of laser levels. Specifically, few studies have utilized empirical data to inversely update FEA models for the construction of a reliable digital baseline model capable of accurate prediction.

To bridge this research gap, this paper proposes an integrated framework combining Pulse-Window Software Lock-in (PWSL) sensing with a data-driven model updating strategy. Initially, a Finite Element Analysis (FEA) model is established to perform dynamic simulations. Subsequently, to overcome the limitations of traditional embedded measurements, a PWSL algorithm featuring a “micro-scale high-speed sampling and macro-scale PC demodulation” architecture is designed, achieving high-fidelity reconstruction of micro-amplitude decay vibrations under strong noise conditions. Finally, based on the precise damping parameters identified via PWSL, the energy dissipation discrepancy in the simulation is quantified, and the FEA model is iteratively updated by introducing an equivalent friction coefficient. This study not only presents a low-cost, high-precision dynamic measurement solution but also provides a reliable basis for the digital design and performance optimization of precision pendulum instruments.

## 2. Theoretical Modeling and Measurement System

### 2.1. Dynamic Model of the Self-Leveling Mechanism

**Figure 1:**
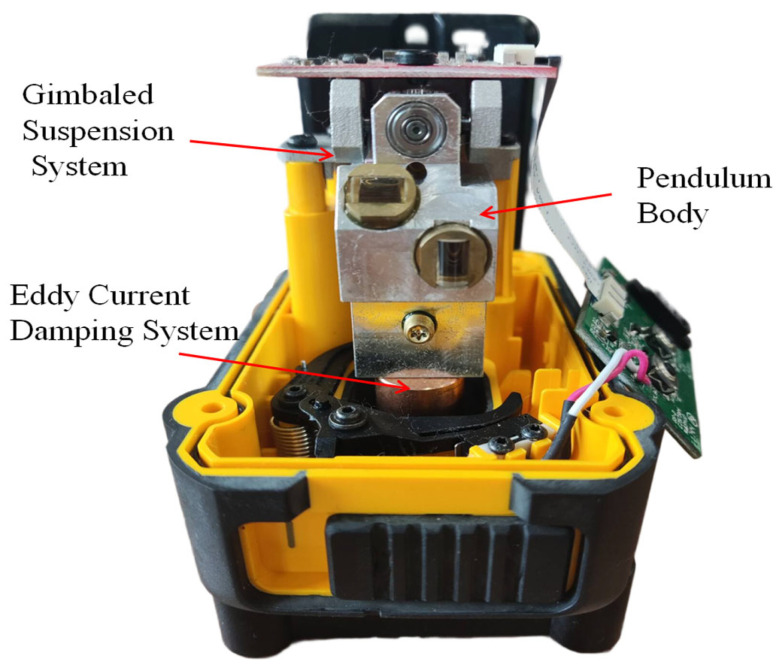
Photograph of the self-leveling mechanism inside the laser level.

The core component of the laser level is the self-leveling mechanism. Structurally, it is essentially a cross-axis suspended pendulum equipped with a magnetic eddy current damping system, primarily consisting of the Pendulum Body, the Gimbaled Suspension System, and the Magnetic Eddy Current Damping System. The physical structure is shown in Figure 1.

The Pendulum Body carries the laser emission module and utilizes gravity to achieve automatic alignment with the plumb line. Supported by rolling bearings, the Gimbaled Suspension System enables two-degree-of-freedom (2-DOF) oscillation in two orthogonal planes; within a small angular range, its motion trajectory approximates that of a spherical pendulum. The Magnetic Eddy Current Damping System, realized by permanent magnets mounted on the base, ensures rapid stabilization of the pendulum following external disturbances.

Ideally, the motion of the self-leveling mechanism constitutes a complex two-degree-of-freedom (2-DOF) coupled system. However, to identify the dynamic characteristic parameters in the direction of the primary disturbance response, the physical system is simplified in this study. Under the assumption of small-angle oscillation, the pendulum body is regarded as an ideal rigid body, and its motion is equivalent to a single-degree-of-freedom (1-DOF) physical pendulum system. This system can be described by a classical second-order linear differential equation, where the two key parameters characterize the dynamic properties. The undamped natural frequency *ω*_n,eq_ and the damping ratio *ζ*_eq_ are defined as follows [12]:

(1) $$\omega_{n,\mathrm{eq}}=\sqrt{\frac{k_{\mathrm{eq}}}{J_{\mathrm{eq}}}}=\sqrt{\frac{\left(m_{\mathrm{eq}}\cdot g\cdot L_{c,\mathrm{eq}}\right)}{J_{\mathrm{eq}}}}$$

(2) $$\zeta_{\mathrm{eq}}=\frac{c_{\mathrm{eq}}}{2\cdot \sqrt{J_{\mathrm{eq}}\cdot k_{\mathrm{eq}}}}$$

where *J*_eq_ and *m*_eq_ denote the equivalent moment of inertia and the total mass of the system, respectively; *L*_c,eq_ represents the distance from the center of rotation to the center of mass; and *g* is the gravitational acceleration. Furthermore, *k*_eq_ signifies the equivalent torsional stiffness induced by gravity, while *c*_eq_ stands for the equivalent viscous damping coefficient of the system.

**Figure 2:**
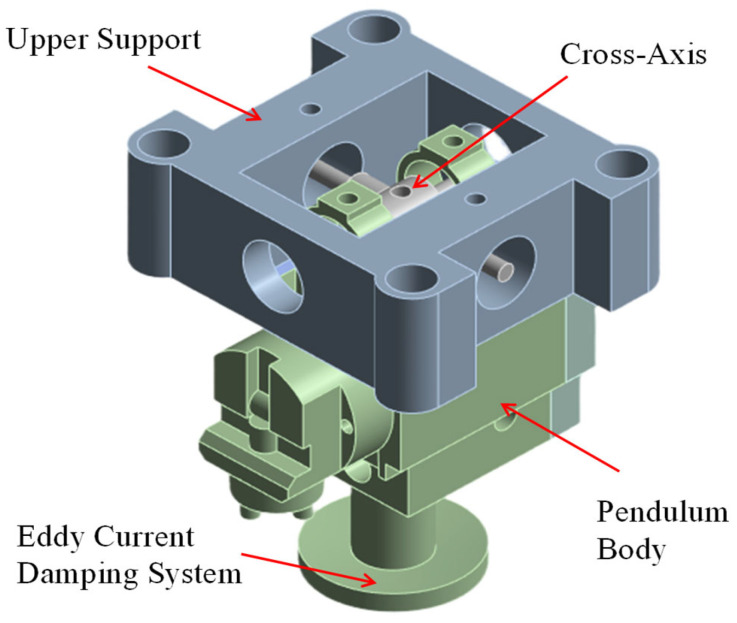
The three-dimensional finite element model of the self-leveling mechanism.

To establish a theoretical baseline, a 3D model was constructed based on the actual dimensions of the prototype and imported into ANSYS Workbench (Version 2024 R1, ANSYS, Inc., Canonsburg, PA, USA)for rigid body dynamics analysis. To focus on the core macroscopic dynamic behaviors and improve computational efficiency, the model was simplified by neglecting minute features, such as fastening screws and circuit boards, that have a negligible impact on the overall inertial properties [13]. Furthermore, physical bearings were substituted with ideal kinematic joints. The final three-dimensional model utilized for simulation is illustrated in Figure 2, which primarily consists of three core components: the Upper Support, the Cross-Axis, and the Pendulum Body.

**Figure 3:**
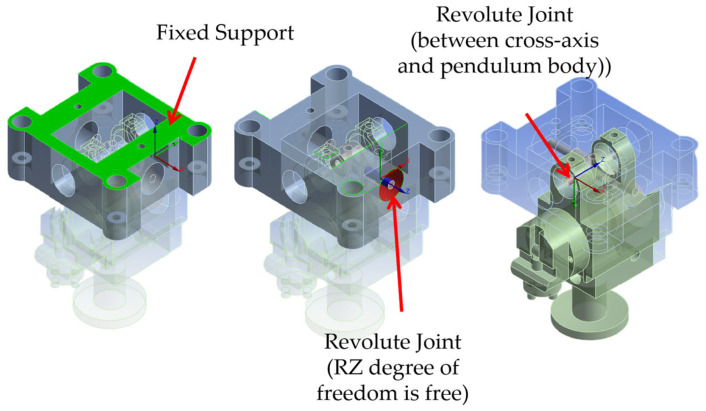
Boundary conditions and kinematic joint definitions in the FEA model.

In the model, all components were defined as rigid bodies. The pendulum body and the support were assigned the material properties of aluminum alloy and stainless steel, respectively (with densities of 2.77 × 10^−6^ kg/mm^3^ and 7.75 × 10^−6^ kg/mm^3^) [14], and the mesh was generated using a default element size of 1 mm. The connection relationships and boundary conditions are illustrated in Figure 3. A fixed support was applied to the upper support to simulate its rigid connection with the instrument housing. Furthermore, revolute joints were established between the upper support and the cross-axis, as well as between the cross-axis and the inner pendulum body, to simulate the two-degrees-of-freedom (2-DOF) rotational characteristics of the mechanism.

To simulate the free decay process of the system, loads, damping, and initial conditions were imposed on the model. Standard Earth gravity (9806.6 mm/s^2^ along the -Y axis) was applied to serve as the source of the system’s restoring torque. An instantaneous tangential velocity of 30 mm/s was applied to the tip of the pendulum body as the initial excitation, ensuring that the oscillation decay remained within realistic operational limits. The total damping effect of the system, primarily derived from magnetic eddy current damping, was implemented by applying equivalent torsional damping to each of the two revolute joints [15]. To quantify the discrepancy between the theoretical model and physical reality, the system damping accounted solely for the theoretical value generated by the eddy current effect, while bearing friction was temporarily neglected. Based on empirical formulas for eddy current damping and the structural parameters of the prototype [16], the equivalent torsional damping coefficient applied to each revolute joint was determined to be 0.00294 N·mm·s/°.

The total simulation duration was set to 5.0 s, with the initial, minimum, and maximum time steps configured as 0.005 s, 0.001 s, and 0.01 s, respectively. To accommodate potential large rotational displacements, the large deflection option was activated. Upon solving, the displacement–time response curve of the model in the Y-direction was obtained.

**Figure 4:**
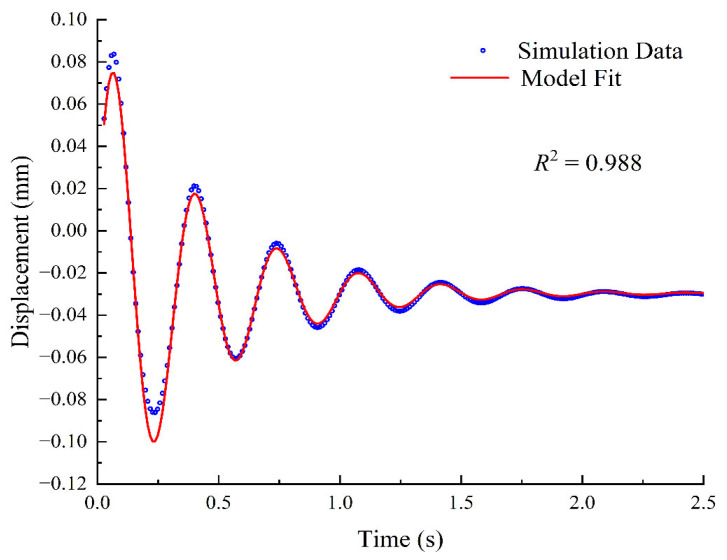
Model fitting of the simulated free-decay response and analysis of residuals.

To extract the dynamic parameters, the theoretical model function was fitted to the simulation curve using the nonlinear least squares method. As shown in Figure 4, the system exhibits characteristic underdamped free decay oscillation, where the amplitude decays exponentially over time. This intuitively reflects the energy dissipation effect of the magnetic eddy current damping torque applied at the joints. The theoretical curve achieves a high degree of coincidence with the simulation data in both phase and amplitude, with a coefficient of determination ($R^{2}$) as high as 0.988. These results quantitatively yielded a theoretical natural frequency of 2.98 Hz and an equivalent damping ratio of 0.126. Furthermore, this validates the rationality of simplifying the complex gimbal self-leveling mechanism into a single-degree-of-freedom (1-DOF) second-order linear system, thereby establishing a reliable theoretical baseline for subsequent experimental identification.

However, the simulation model represents physical predictions solely under idealized conditions. To acquire authentic, high-fidelity dynamic response data, a high-signal-to-noise ratio (SNR) non-contact measurement system based on a Position Sensitive Detector (PSD) was constructed, as detailed in Section 2.2.

### 2.2. Hardware Architecture of the Measurement System

**Figure 5:**
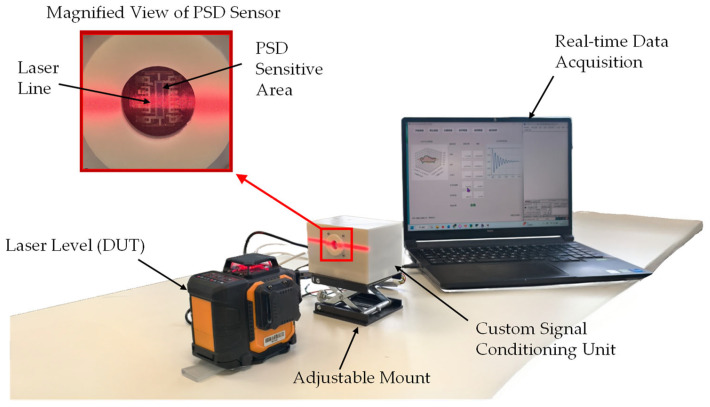
Block diagram and experimental setup of the non-contact measurement system. The system integrates a Position Sensitive Detector (PSD) with a custom signal conditioning unit to capture micro-amplitude vibrations, where the STM32 microcontroller acts as a high-speed data gateway to the PC workstation.

In this study, a high signal-to-noise ratio (SNR) non-contact photoelectric measurement platform was developed to accurately capture the micro-amplitude decay vibrations of the laser level’s self-leveling mechanism. The physical connections and system architecture of the experimental setup are illustrated in Figure 5.

The measurement system primarily comprises three core subsystems. The photoelectric detection front-end employs a Hamamatsu S5609 (Hamamatsu Photonics, Hamamatsu City, Japan) one-dimensional Position Sensitive Detector (PSD) as the core sensing element. In contrast to traditional discrete photodiodes, the PSD leverages the lateral photoelectric effect and possesses a continuous photosensitive surface, enabling sub-micron position resolution and microsecond-level response speeds [17]. The PSD is mounted within a custom 3D-printed shielding enclosure to minimize interference from ambient stray light [18]. The signal conditioning and data acquisition unit incorporates a precision transimpedance amplifier circuit based on the OPA4131 and a 16-bit high-precision ADC (AD7606), with synchronous control and data transmission managed by an STM32F407 microcontroller (STMicroelectronics, Geneva, Switzerland).

Crucially, the STM32 functions solely as a high-speed transparent data gateway; all complex signal processing tasks are executed on the PC workstation. Leveraging the high computational power of the PC, position information is demodulated online in real-time for waveform monitoring, while offline identification is performed on data segments post-acquisition to extract dynamic parameters.

The experiments were conducted in a standard laboratory environment with a controlled temperature of 25 ± 1 °C and a relative humidity of 45 ± 5%. To minimize external electromagnetic interference (EMI), shielded twisted-pair cables were employed for all analog signal transmissions. Furthermore, the inherent narrowband pass characteristic of the proposed PWSL algorithm (locking at 10 kHz) effectively rejects broadband electromagnetic noise and 50 Hz power-line interference, ensuring a high Signal-to-Noise Ratio (SNR) without requiring bulky shielding enclosures.

**Figure 6:**
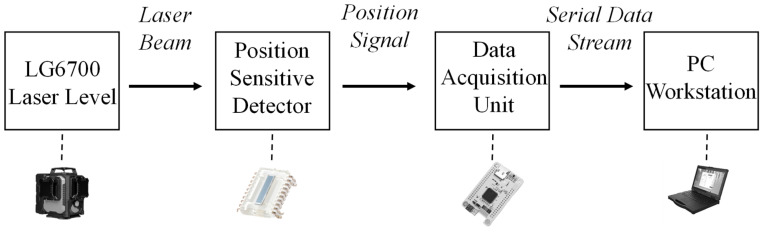
Schematic of the experimental setup for dynamic parameter identification.

The Data Acquisition (DAQ) unit employs a specialized pulse-window sampling architecture. Although the position data update rate transmitted upstream is 100 Hz, the ADC performs high-speed oversampling at a rate of 150 kHz within each sampling cycle to capture the complete 10 kHz carrier waveform of the laser signal. This “micro-scale high-speed acquisition, macro-scale low-speed output” architecture provides sufficient data redundancy to extract weak displacement signals from strong noise backgrounds. The schematic of the signal transmission flow is shown in Figure 6.

To eliminate the inherent non-linear distortion near the edges of the PSD’s effective active area, a static linearity calibration was conducted prior to the dynamic identification experiments. A precision linear translation stage (positioning accuracy < 1 μm) was employed to perform a scanning test of the laser spot over a range of ±3 mm with a step size of 0.1 mm. Subsequently, a regression model based on a 5th-order polynomial was established to compensate for systematic errors. Following calibration, the Root Mean Square Error (RMSE) of the measurement system was reduced from 37.97 μm to 28.44 μm, thereby providing a reliable micron-level precision baseline for the subsequent dynamic modeling.

To accurately extract micro-amplitude decay characteristics from noisy signals, an advanced signal processing strategy is indispensable. Section 2.3 details the Pulse-Window Software Lock-in (PWSL) algorithm, which was specifically designed to address this challenge.

### 2.3. Pulse-Window Software Lock-In Algorithm

**Figure 7:**
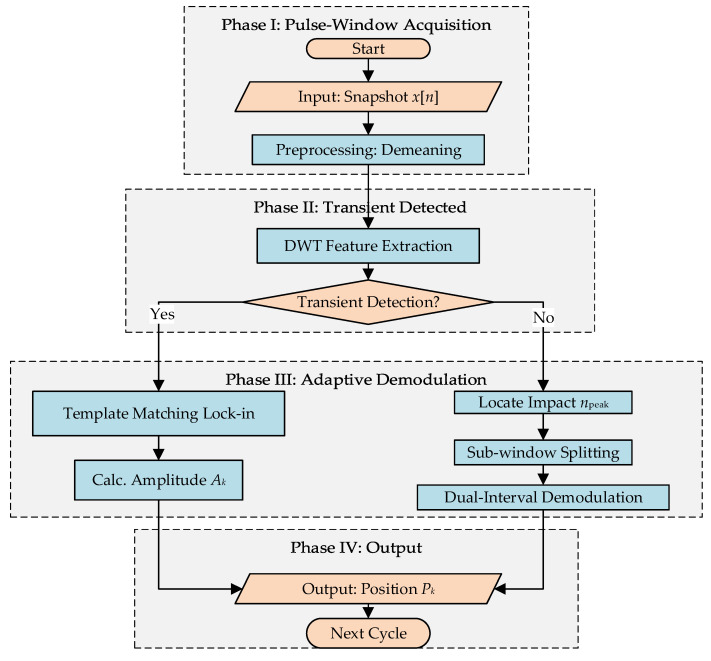
Flowchart of the Pulse-Window Software Lock-in (PWSL) algorithm. The workflow consists of four distinct phases: Pulse-Window Acquisition, Transient Detection, Adaptive Demodulation, and Position Output, enabling intelligent switching between high-SNR extraction and transient capture modes.

To overcome the bandwidth bottlenecks of embedded data transmission and fully leverage the computational power of the PC workstation to address the inflexibility of traditional hardware demodulation, this study proposes a Pulse-Window Sampling-based Wavelet-Enhanced Software Lock-in (PWSL) algorithm. Adopting a “micro-scale high-speed acquisition, macro-scale feature extraction” architecture, the algorithm balances steady-state accuracy with transient response capabilities. The core workflow is illustrated in Figure 7.

The core of the PWSL algorithm comprises two distinct components. The front-end employs a pulse-window sampling strategy designed to alleviate the data throughput bottlenecks inherent in embedded systems. The back-end features a Wavelet-Enhanced Adaptive Software Lock-in workflow. By performing online discrimination of time-frequency characteristics, it intelligently switches between two optimal strategies—“high SNR extraction” and “high temporal resolution localization”—to adaptively achieve high-fidelity and highly robust signal demodulation.

**Figure 8:**
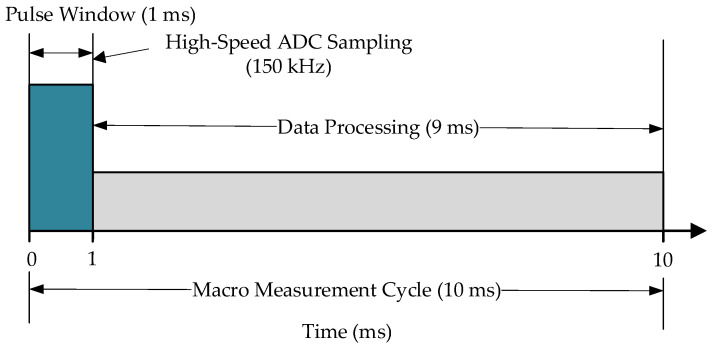
Schematic of the pulse-window sampling timing sequence.

The front-end of the PWSL algorithm employs a short-time pulse-window sampling strategy featuring a non-uniform sampling timing sequence. This approach addresses the issue of massive data redundancy encountered in traditional continuous oversampling due to the 10 kHz carrier characteristics of the laser level, as illustrated in Figure 8.

The system is configured with a macro data update rate of 100 Hz. Within each pulse window period, the ADC executes high-speed sampling, capturing a data snapshot containing *N* points at a sampling rate of 150 kHz. While fully preserving the carrier waveform characteristics, this strategy reduces the average data throughput by over 90%. Consequently, it effectively overcomes the real-time data transmission bottleneck in embedded systems associated with high sampling rates, ensuring that raw waveform data are successfully transmitted to the PC workstation for downstream processing.

The back-end of the PWSL algorithm employs an adaptive demodulation mechanism featuring a “discrimination-first, processing-subsequent” strategy to address the characteristics of non-stationary signals in industrial environments.

First, transient event discrimination is performed. The pre-processed snapshot is subjected to single-level decomposition using the db4 wavelet basis [19]. The maximum modulus of the first-level detail coefficients is calculated as the transient energy indicator. If this value exceeds an adaptive threshold derived from the Universal Threshold Rule, the current window is identified as containing a transient shock, triggering the high-resolution processing mode; otherwise, it is processed as a steady-state signal.

For steady-state signals, a software lock-in amplification technique based on physical template matching is adopted. Cross-correlation is performed between the signal and a pre-calibrated single-cycle physical template to achieve phase locking. Subsequently, the amplitude is extracted via differential integration based on the “light/dark” intervals defined by the template. Leveraging the orthogonal correlation between the signal and the template, this method effectively suppresses common-mode noise and baseline drift.

Conversely, for transient signals, the algorithm utilizes wavelet coefficients to localize the abrupt change point (breakpoint), segmenting the current 1 ms window into two sub-intervals for independent demodulation. This enables the system to output two position data points within a single sampling cycle, achieving sub-millisecond dynamic response capture and effectively resolving the phase lag issue associated with traditional low-pass filters.

Through this workflow, the algorithm realizes automatic switching between the two modes, ensuring steady-state measurement precision while fully preserving the time-domain characteristics of transient shocks. Having completed the theoretical modeling, hardware implementation, and algorithm design, Section 3 will proceed to validate the algorithm’s noise immunity and perform dynamic parameter identification.

## 3. Experimental Identification

### 3.1. Verification of Algorithm Noise Immunity

**Figure 9:**
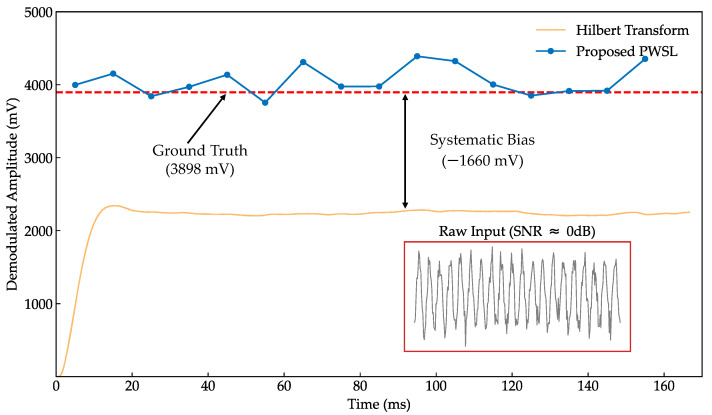
Performance comparison under strong Gaussian white noise. The proposed PWSL method (blue line) accurately recovers the ground truth amplitude, whereas the traditional Hilbert Transform (yellow line) exhibits a significant systematic bias (−1660 mV) due to the rectification error of superimposed noise energy.

To comprehensively evaluate the signal extraction capability of the PWSL algorithm within complex noise environments, a semi-physical simulation experiment was conducted to benchmark its noise immunity against the traditional Hilbert Transform demodulation method. An interference-free experimental laser signal served as the amplitude Ground Truth, onto which high-intensity random Gaussian white noise was injected to simulate typical harsh operating conditions. The comparison of the demodulation results is illustrated in Figure 9, and the quantitative performance metrics are presented in Table 1.

The comparative results reveal that the traditional Hilbert method, due to the inherent nature of its RMS-based energy detection, suffers from significant systematic bias under strong noise conditions. This deviation stems from the superposition of the DC component of the noise, rendering the method virtually ineffective under severe interference. In contrast, the PWSL method developed in this study integrates the principles of common-mode rejection and matched filtering, achieving high-fidelity extraction of the true amplitude of weak signals from strong random noise. Although the PWSL output retains certain random fluctuations in the time domain, these are effectively eliminated through subsequent position normalization, ensuring that the mean value converges highly to the ground truth.

**Figure 10:**
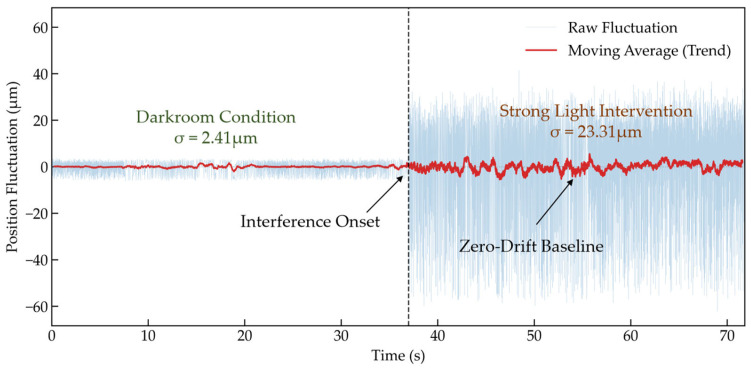
Time-domain response of position fluctuation before and after strong light intervention. Although the fluctuation amplitude increases due to shot noise under intense lighting, the mean position value remains stable around the zero-drift baseline without saturation, demonstrating the algorithm’s effectiveness in rejecting DC drift and 50 Hz power-line interference.

While the Gaussian white noise simulation presented above provides a theoretical baseline for random noise rejection, realistic industrial environments often contain complex non-Gaussian interference. To strictly verify the system’s robustness under these conditions, a physical experiment involving 50 Hz stroboscopic interference and direct strong light exposure was conducted. This setup specifically targets the two most common industrial noise sources: power-line frequency flicker (periodic noise) and high-intensity ambient light (baseline drift), which are not fully captured by standard Gaussian models. To this end, the time-domain response of the measurement system was tested during a transition from a “darkroom” condition to a “strong stroboscopic interference” scenario (superimposed with 50 Hz stroboscopic noise and direct intense light). The results are illustrated in Figure 10.

At the onset of strong interference, although the shot noise introduced by ambient light caused a slight increase in the fluctuation amplitude of the measurement data, the mean value remained tightly oscillating around the baseline. Crucially, no monotonic drift or saturation clipping phenomena were observed. This demonstrates that the PWSL algorithm, through its differential sampling mechanism based on light/dark windows defined by the physical template, effectively cancels out the DC component induced by intense background light at the physical level. Simultaneously, the software lock-in process successfully isolates the 50 Hz power-line flicker noise.

**Figure 11:**
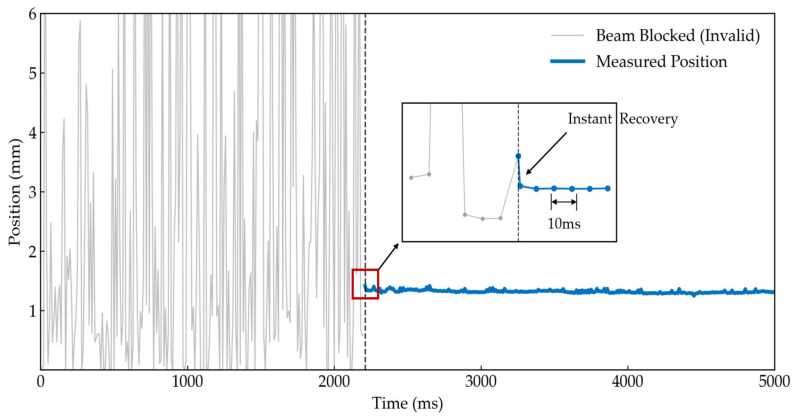
Step response characteristics of the optical measurement system. The system resolves the steady-state position within a single 10 ms sampling cycle immediately after the optical signal recovers, confirming its zero-lag single-frame locking capability essential for dynamic capture.

Finally, to verify the measurement system’s capability to capture the rapid transient oscillations of the self-leveling mechanism, an optical path step recovery experiment was designed. A near-ideal optical step signal was generated by rapidly withdrawing a baffle that blocked the laser beam. The response time of the system was observed, with the results presented in Figure 11.

Figure 11 clearly illustrates the discrete-time response characteristics of the system. Upon the recovery of the optical signal from occlusion, the system completes position resolution and outputs the steady-state value within the current single 10 ms sampling cycle. This demonstrates that the system is free from cumulative filtering delay and possesses single-frame locking capability. This Zero-Lag characteristic is paramount for measuring dynamic self-leveling parameters. It ensures phase fidelity in the acquired decay curves, thereby circumventing the impact of phase lag—typically introduced by traditional moving average algorithms—on the accuracy of damping ratio identification.

### 3.2. Parameter Identification Based on Dual Sliding Window Scanning

**Figure 12:**
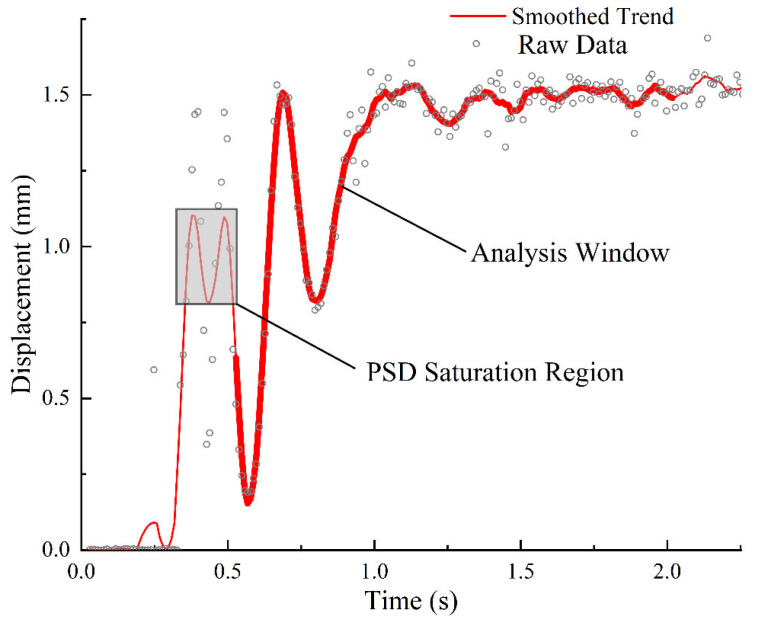
Identification of the saturation region and selection of the analysis window in raw data.

Utilizing the measurement system constructed in Section 2, an initial angular displacement perturbation was applied to the self-leveling mechanism to induce free decay oscillation. Through demodulation using the PWSL algorithm, the complete time-domain position response signal was recorded. Ultimately, discrete data points characterizing the system’s dynamic response were obtained, and a typical envelope curve is illustrated in Figure 12.

It can be observed from the raw position signal envelope that during the initial excitation phase (approximately 0–500 ms), the large-amplitude oscillation of the pendulum causes the laser spot to exceed the linear measurement range of the PSD sensor. Consequently, the non-linear data within this saturation region is rendered invalid. Direct fitting of the data over the entire duration would inevitably lead to significant identification errors. Furthermore, given the slight non-stationarity of the system, manual selection of the data truncation window would introduce subjective bias.

To address this, a Dual Sliding Window Scanning (DSWS) strategy is proposed in this study. Instead of relying on a single fixed window, this strategy employs nested loops to iterate through all possible start points *t*_start_, and end points *t*_end_. Subsequently, least squares fitting is performed on the data within each sub-window based on the second-order damped oscillation model:

(3) $$P(t)=P_{\mathrm{stable}}+A_{0}\cdot e^{-\delta t}\cdot \sin(\omega_{d}t+\psi )$$

where *P*_stable_ represents the steady-state equilibrium position, and *A*_0_ and *ψ* denote the initial displacement amplitude and phase, respectively. The critical parameter *δ* is the decay rate, and *ω*_d_ represents the damped natural frequency.

Upon extracting *δ* and *ω*_d_ through the fitting algorithm, the system’s equivalent natural frequency (*ω*_n,eq_) and equivalent damping ratio (*ζ*_eq_) can be calculated using the following relationships:

(4) $$\omega_{n,\mathrm{eq}}=\sqrt{\omega_{d}^{2}+\delta^{2}}$$

(5) $$\zeta_{\mathrm{eq}}=\frac{\delta }{\omega_{n,\mathrm{eq}}}$$

The DSWS strategy enables the objective extraction of dynamic parameters that best represent the system’s intrinsic characteristics, effectively eliminating the bias arising from the arbitrariness of manual window selection [20].

**Figure 13:**
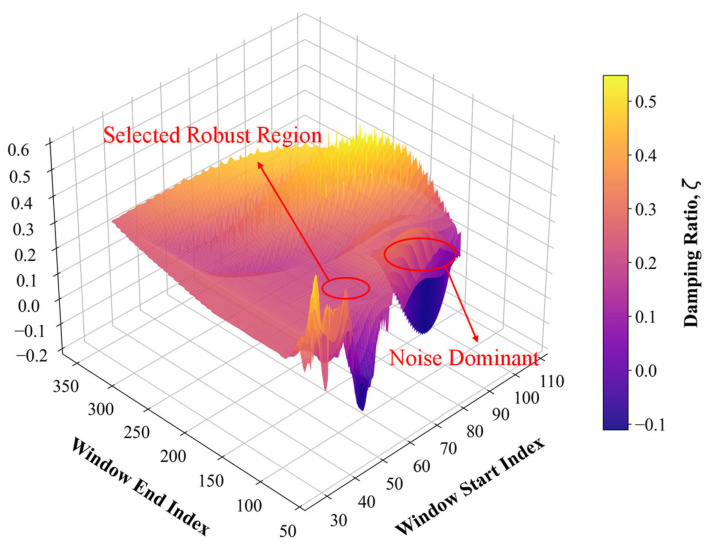
Stability surface of the identified damping ratio varying with analysis window parameters.

By employing scanning rules based on nested loops, a series of analysis windows with varying start and end indices are generated, and fitting is performed on the data within each individual window. Finally, through statistical analysis of all fitting results demonstrating high goodness-of-fit, the optimal parameters within the convergence region are selected as the definitive identification output for the system [21]. Figure 13 clearly demonstrates the convergence trend of the identification results as a function of the analysis window variations.

**Figure 14:**
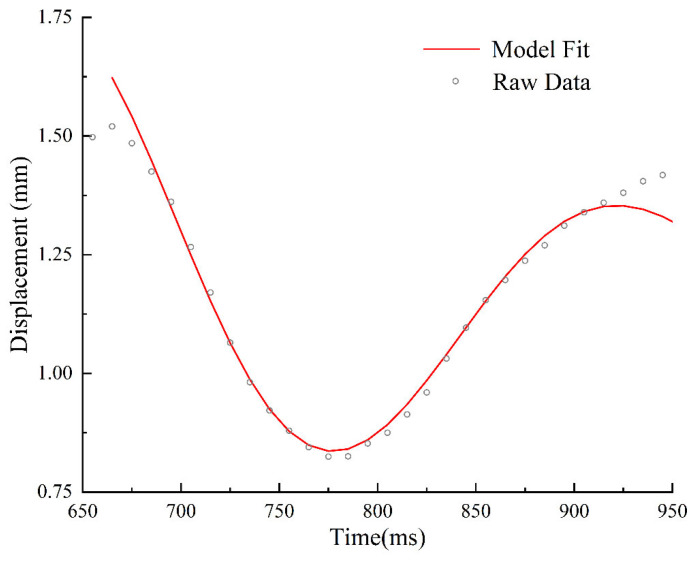
Equivalent linear model fitting results for the selected robust window. The red solid line represents the theoretical curve fitted using the second-order underdamped oscillation model, which shows excellent agreement with the experimental raw data.

Based on the statistical analysis, the optimal fitting interval was determined to be 650 ms to 950 ms. The final parameters were derived by calculating the mean of all fitting results within this interval that exhibited a goodness-of-fit $R^{2}$ exceeding 0.99. The final fitting performance is illustrated in Figure 14.

To verify the reliability of the identification method and evaluate the repeatability of the system response, three independent dynamic response experiments were conducted. The data from each experiment was analyzed using the aforementioned Window Scanning Method. The results of all successful fittings that met the high goodness-of-fit criteria were statistically averaged, and the final outcomes are presented in Table 2.

A final statistical analysis was performed on the three sets of results listed in Table 2. The system’s average natural frequency was determined to be 2.861 ± 0.160 Hz, and the average damping ratio was 0.1435 ± 0.0087 (represented as mean ± sample standard deviation). Notably, the extremely small standard deviation of the damping ratio (0.0087) demonstrates the high consistency of the DSWS algorithm in identifying the system’s equivalent damping characteristics.

To rigorously quantify the reliability of the identified dynamic parameters, an uncertainty propagation analysis was performed. Given the nonlinearity of the decay model, a Monte Carlo simulation approach was adopted to map the sensor measurement error to the identified parameter space. Based on the static linearity calibration results in Section 2.2, the random noise of the PSD sensor was characterized by a Root Mean Square Error (RMSE) of 28.44 μm. This RMSE value represents the experimental measurement error. Regarding the reference values, since invasive contact sensors would alter the system’s mass and damping characteristics, the theoretical decay curve fitted with high precision (*R*^2^ > 0.99) serves as the ‘Ground Truth’ baseline.

**Figure 15:**
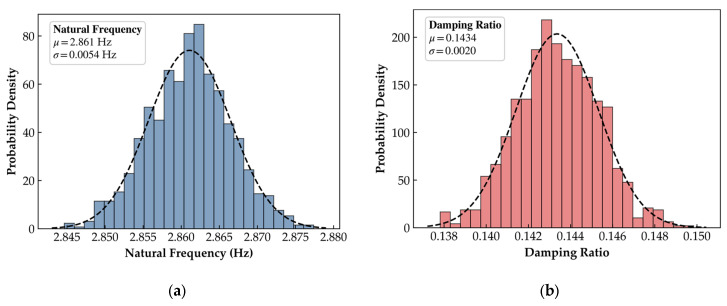
Uncertainty propagation analysis results based on Monte Carlo simulation (*N* = 1000): (**a**) Probability density distribution of the identified natural frequency; (**b**) Probability density distribution of the identified damping ratio. The black dashed lines represent the Gaussian fit curves.

Consequently, Gaussian white noise with a standard deviation of *σ*_noise_ = 0.02844 mm was superimposed onto the ideal theoretical decay response curve (*f_n_* = 2.861 Hz, *ζ* = 0.1434), and a total of 1000 independent simulation trials were executed. The statistical distribution of the identification results is illustrated in Figure 15.

It can be observed that the identified parameters strictly follow a Gaussian normal distribution, indicating that the algorithm possesses unbiased estimation characteristics. Quantitatively, the propagated standard deviation for the natural frequency is *σ_f_* = 0.0054 Hz, representing a relative uncertainty of only 0.19%. Similarly, the standard deviation for the damping ratio is *σ_ζ_* = 0.0020, corresponding to a relative uncertainty of 1.39%.

These results demonstrate that the impact of sensor measurement noise on the final identification accuracy is minimal. The proposed PWSL sensing system and the DSWS identification strategy exhibit high robustness against noise interference, ensuring that the experimentally derived parameters are statistically significant and reliable for the subsequent model updating process.

## 4. Results Analysis and Model Updating

The statistically averaged values obtained from experimental identification were cross-compared with the FEA simulation predictions presented in Section 2. The results are summarized in Table 3.

The comparative analysis of the data in Table 3 reveals that the experimentally identified average natural frequency of the system is highly consistent with the simulation prediction. Considering that the bearings were simplified as ideal hinges and the mass influence of minor attachments (such as cables) was neglected during the simulation modeling, this result validates the accuracy of the established finite element model regarding macroscopic mass distribution and structural stiffness characteristics.

However, a relative error of 14.34% persists between the experimentally identified average damping ratio and the simulated value [22]. This discrepancy is primarily attributed to the exclusion of secondary energy-dissipating factors, such as non-linear Coulomb friction at the cross-bearings and air viscous damping [23]. Nevertheless, the extremely low standard deviation of 0.0087 observed across three independent repeated experiments confirms the high reliability of the DSWS algorithm in identifying the system’s equivalent damping characteristics.

To eliminate the aforementioned “virtual–real discrepancy” and establish a high-fidelity finite element model, an iterative model updating strategy based on experimental data was implemented, focusing specifically on damping parameters. Parametric scanning and fine-tuning of the joint’s torsional damping coefficient were performed within the ANSYS Transient Structural module to compensate for the unmodeled friction losses [24]. Through iterative optimization, when the total damping coefficient was adjusted to 0.00340 N·mm·s/°, the damping ratio of the updated model increased to 0.1449, reducing the relative error to 0.97%.

**Figure 16:**
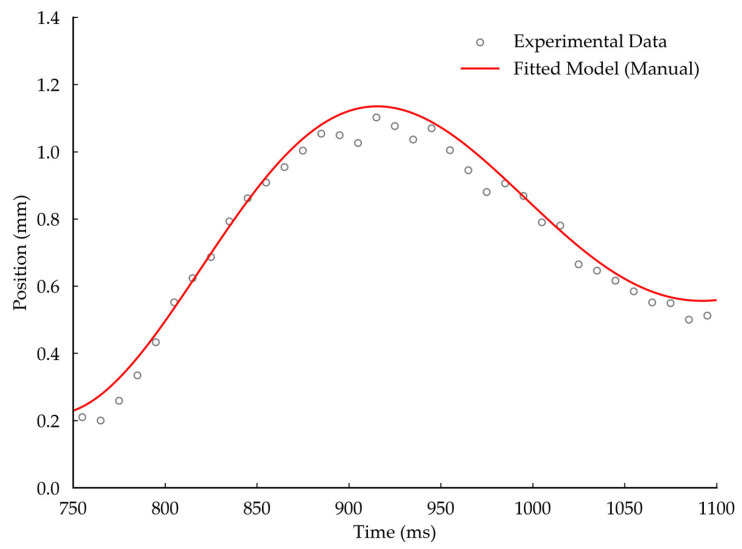
Time-domain comparison between the updated FEA simulation and experimental measurement.

As illustrated in Figure 16, the updated simulation decay curve demonstrates a high degree of coincidence with the experimental measurement curve in terms of time-domain phase synchronization and amplitude decay rate. This provides a reliable basis for the structural optimization of such precision pendulum-type instruments.

It is worth noting that the mechanical friction in the bearing may change over the instrument’s lifecycle due to wear. Since the proposed updating strategy identifies the equivalent damping ratio (*ζ*) directly from the experimental decay data, any increase in friction caused by wear will be captured as an increase in the identified *ζ*. In the model updating process, this identified damping variation is mapped to the equivalent friction torque parameters in the finite element model. This ensures that the simulation remains physically consistent with the actual wear state, maintaining prediction accuracy over time.

## 5. Conclusions

Addressing the challenges in dynamic modeling of the self-leveling mechanism of laser levels, this study proposes a high-fidelity modeling framework that integrates Pulse-Window Software Lock-in sensing with a model updating strategy. The main conclusions are summarized as follows:

1. High-Precision Measurement and Parameter Identification Achieved: The proposed PWSL algorithm, combined with the DSWS strategy, achieved high-fidelity reconstruction of transient decay signals within strong noise environments. The system identification results yielded a natural frequency of 2.861 ± 0.160 Hz and a damping ratio of 0.1435 ± 0.0087, demonstrating high robustness.
2. Quantification of Finite Element Model Errors: The relative error between the experimentally identified natural frequency and the initial FEA prediction was only 4.22%, confirming the accuracy of the model regarding mass distribution and stiffness characteristics. Conversely, the 14.34% relative error in the damping ratio quantitatively revealed the energy dissipation prediction bias inherent in standard finite element models when micro-friction in bearings is neglected.
3. Model Updating and Digital Twin Construction: Based on experimental data, a model updating and digital twin construction process was implemented. By introducing an equivalent friction damping coefficient to inversely update the FEA model, the final model damping prediction error was reduced to <1%, successfully constructing a High-Fidelity Digital Benchmark of the laser level’s self-leveling mechanism.
4. Limitations and Future Work: A limitation of this study is that validation was conducted based on a single-degree-of-freedom model under controlled ambient temperature. Future work will incorporate two-dimensional cross-coupling effects and thermal–magnetic coupling mechanisms to establish a multi-physics nonlinear model, thereby enhancing the engineering applicability of the method under extreme variable temperature conditions.

In summary, this study not only presents a low-cost, high-precision non-contact dynamic measurement scheme but also establishes a closed-loop methodology spanning from “signal processing” to “physical modeling.” This framework provides a reliable theoretical basis for the structural optimization and performance prediction of precision pendulum-type instruments.

## Figures and Tables

**Table 1:** Performance comparison under injected strong white noise (σ = mV).

Performance Metric	PWSL	Traditional Hilbert	Comparison
Absolute Accuracy Error (mV)	27.11	1728.16	63.7-fold improvement
Measurement Standard Deviation (mV)	213.65	300.39	1.4-fold improvement

**Table 2:** Summary of final identification results from repeatability experiments.

Experiment No.	1	2	3
Average Natural Frequency (Hz)	2.6896	3.0079	2.8847
Average Damping Ratio	0.1496	0.1336	0.1473

**Table 3:** Comparison between simulated prediction and experimental identification.

Parameter	FEA Prediction	Experimental Identification	Relative Error
Natural Frequency (Hz)	2.987	2.861	4.22%
Damping Ratio	0.1255	0.1435	14.34%

## Data Availability

The original contributions presented in this study are included in the article. Further inquiries can be directed to the corresponding author.
